# Alterations of the Blood in Syphilis

**Published:** 1898-02

**Authors:** 


### ALTERATIONS OF THE BLOOD IN SYPHILIS.

Justus (British Journal of Dermatology) has made a special study of the anemia, well recognized as occurring early in syphilitic infection. He finds that the percentage of hemoglobin undergoes regular fluctuations commencing with the appearance of general glandular enlargement. It decreases slowly and steadily to its limit at the time of the secondary accidents. If the patient remains untreated, it increases to its normal level in health with their subsidence. Syphilis therefore destroys the hemoglobin which is built up again as the patient recovers spontaneously. With the administration, however, of a fairly large dose of mercury, the percentage drops immediately, to recover in a number of days proportionate to the virulence of the disease and the state of the patient. With every succeeding dose the drop is less marked until after a thorough course of mercurials the amount is greater than before the administration of the drug. The subsidence of the early accidents is coincident with the increase in resisting power of the hemoglobin, the disease itself causing temporary diminution of that power as regards mercury. * * * Valerio from a study of five cases reaches practically the results of Justus. He says that the alkalinity, density and the chlorids in the blood are diminished in addition. As in previous researches, the author states that the globular resistance of the blood is proportionate to its richness in the chlorids of sodium and potassium. The biniodid of mercury, all things being equal, is more powerful in restoring the blood to its normal state than the protiodid.

---

Solberg is reported in the *Deutsche Medizinal-Zeitung* of August fifth, 1897, as using, in a case of pneumonia with severe pain in the side in which he could not resort to the injection of morphine, a strip of adhesive plaster, and the result was surprisingly prompt; as in cases of fracture of a rib. He has since employed the plaster in six other cases of severe pain in the side occurring in the course of pneumonia. In four of them, in which the inflammation was in the lower lobe, the improvement was notable. In another case, in which the "stitch" was really in the scapular region, alleviation was effected by applying the strip of plaster directly beneath the axilla. In the sixth case, in which the "stitch" was not severe and the strip was removed at the end of a day because the patient felt a little constrained by it, it was again applied at the patient's request. Even the dyspnœa and the cough seemed to be mitigated, according to Solberg's observation and the patient's own statements. The strip used was of American adhesive plaster, not more than an inch and a half wide, applied as in cases of fractured ribs.

---

It has been a regulation in Hamburg, for twenty-seven years, that every prostitute confined at the public hospital must bring her child to the city physician every month or oftener for examination, until it is a year old. S. Werner publishes a report of the results in the *Woch. f. prak. Derm.*, Vol. XXIV. Nos. 4 and 5, which is an important contribution to the study of hereditary syphilis. The mortality among the children is high, 63.5 per cent. from syphilitic mothers; 57 per cent. from non-syphilitic. Several instructive instances are related of inheritance of post-conception infection, and one of the still disputed *choc en retour*. Tardy symptoms after the first year were seldom noted. Four healthy and five diseased children were born in nineteen cases of simultaneous conception and infection, the rest aborted. Fourteen children inherited syphilis in thirty-one cases of tertiary disease. The effect of treatment before and during pregnancy is also studied.—*Cbl. f. Chir.*, Aug. 4.—*N. Y. Polyclinic.*

